# House dust mite (Derp 10) and crustacean allergic patients may be at risk when consuming food containing mealworm proteins

**DOI:** 10.1186/2045-7022-3-S3-P48

**Published:** 2013-07-25

**Authors:** KC Verhoeckx, S van Broekhoven, M Gaspari, SC de Hartog-Jager, G de Jong, H Wichers, E van Hoffen, G Houben, AC Knulst

**Affiliations:** 1 TNO, Zeist, the Netherlands; 2 Dermatology/Allergology, University Medical Center Utrecht (UMCU), Utrecht, the Netherlands; 3 Utrecht Center for Food Allergy (UCFA), Utrecht, the Netherlands; 4 Laboratory of Entomology, Wageningen University and Research Centre, Wageningen, the Netherlands; 5 Dipartimento di Medicina Sperimentale e Clinica, Università "Magna Græcia" di Catanzaro, Catanzaro, Italy; 6 Food & Biobased Research, Wageningen University and Research Centre, Wageningen, the Netherlands

## Background

Due to the imminent growth of the world population, shortage of protein sources for human consumption will arise in the near future. Alternative and sustainable protein sources (e.g. insects and algae) are now being explored for the production of food and feed. In this project the safety of mealworm (*Tenebrio molitor* L.) proteins for human consumption was tested according to the European Food Safety Authority (EFSA) [1] guidelines for allergenicity risk assessment of genetically modified organisms (GMO).

## Methods

Different mealworm protein fractions (soluble and insoluble) were prepared, characterized, and tested for cross-reactivity using IgE from patients with an inhalation or food allergy to closely related species (house dust mite and crustacean) according to the phylogenetic tree, using immunoblotting and indirect basophil activation. Furthermore, the stability was investigated using an *in vitro* pepsin digestion test.

## Results

IgE from both house dust mite and crustacean allergic patients cross-reacted with proteins in mealworm. This cross-reactivity was functional, as shown by the induction of basophil activation. The cross-reactive proteins were identified as tropomyosin and arginine kinase, which are well known allergens in lobster, shrimp and house dust mite. These proteins were mildly stable in the pepsin stability test.

## Conclusion

Based on these cross-reactivity studies, house dust mite and crustacean allergic patients may be at risk when consuming food containing mealworm proteins.

## Disclosure of interest

None declared.
